# Relation between augmentation index and adiponectin during one-year metformin treatment for nonalcoholic steatohepatosis: effects beyond glucose lowering?

**DOI:** 10.1186/1475-2840-11-61

**Published:** 2012-06-07

**Authors:** Marina Shargorodsky, Elena Omelchenko, Zipora Matas, Mona Boaz, Dov Gavish

**Affiliations:** 1 Department of Endocrinology, Wolfson Med Center, POB 5, Holon, 58100, Israel; 2 Department of Medicine, Wolfson Med Center, POB 5, Holon, 58100, Israel; 3 Department of Biochemistry, Wolfson Med Center, POB 5, Holon, 58100, Israel; 4 Epidemiology and Research Unit, Wolfson Med Center, POB 5, Holon, 58100, Israel; 5 Sackler School of Medicine, Tel Aviv University, Tel Aviv, Israel

## Abstract

**Background:**

Insulin resistance (IR) is the major driving force behind development and progression of atherosclerosis in patients with nonalcoholic fatty liver disease (NAFLD). Therefore, correction of IR is a relevant therapeutic target.

We performed the current trial to evaluate whether 12- month metformin therapy improves vascular stiffness in patients with NAFLD and to assess if this improvement is associated with change in glucose control, insulin resistance or circulating adiponectin.

**Methods:**

In randomized, placebo controlled study, 63 patients with NAFLD were assigned to one of two groups: Group 1 received daily metformin; Group 2 received placebo. Central aortic augmentation index (AI) was performed using SphygmoCor (version 7.1, AtCor Medical, Sydney, Australia) at baseline, at 4-and 12-month treatment period. Metabolic parameters, insulin resistance markers and serum adiponectin levels were determined.

**Results:**

In placebo group: AI did not improve during the treatment period. Liver function and adiponectin levels did not change during the study.

In multiple linear regression analysis, the independent predictors of arterial stiffness improvement were metformin treatment and increase in circulating adiponectin levels.

Among metformin treated patients: AI decreased significantly during the study. ALP and ALT decreased during initial 4-month treatment period, however raised to the pretreatment levels after 12 months. Serum adiponectin level tended to increase during treatment period with metformin.

**Conclusions:**

Metformin treatment was associated with significant decrease in AI during one year treatment in NAFLD patients. These beneficial vascular effects was associated with exposure to metformin per se as well as change in adiponectin levels suggesting that metformin may mediate its vascular effects via glicemic control-independent mechanisms.

**Trial registry:**

no: NCT01084486

## Background

Insulin resistance is one of the major driving forces behind development and progression of atherosclerosis in patients with nonalcoholic fatty liver disease (NAFLD) [1-4]. Metformin is a potent insulin sensitizer, and, as such, has the potential to alleviate the cardiovascular damage caused by insulin resistance. Several clinical studies have shown significant improvement in insulin resistance and liver function as well as positive histological changes in patients treated with metformin [5,6]. We have previously reported that 4 months metformin treatment was associated with a significant improvement in arterial stiffness in patients with nonalcoholic fatty liver disease [7]. Metformin has beneficial effects on glucose control as well as traditional cardiovascular risk factors and is associated with decreased cardiovascular morbidity and mortality in diabetic patients [8]. However accumulating evidence suggests that metformin may affect the risk of atherothrombotic disease through mechanisms other than lowering glycemia, possibly by vasculoprotective effects [9-11]. Recently published data indicate that metformin treatment is associated with a significant increase in serum adiponectin [12], which plays an important role in insulin sensitivity, inflammation, lipid metabolism and atherogenesis [13-15].

Since the long-term vascular impact of treatment, which improves insulin sensitivity in patients with NAFLD, has not been investigated, the present study was designed to determine the long-term vascular effect of metformin treatment in this population. Central aortic augmentation index, determined invasively, has been reported to be a strong, independent risk marker for premature coronary artery disease [16]. Although AI is influenced by several parameters, such as age, heart rate, SVR, left ventricular hypertrophy, endothelial function, this parameter is considered to be reliable and valid measure of arterial stiffness, and significantly and independently associated with target organ damage [17] as well as cardiovascular morbidity and mortality [18,19]. Measurement of arterial stiffness may serve not only as cardiovascular risk assessment [20] but also as an indicator of treatment benefit [21].

Two questions were addressed: (1) Whether 12- month metformin therapy improves vascular stiffness in patients with NAFLD. (2) Whether effect of long-term metformin treatment on arterial stiffness is dependent of glycaemic control, insulin resistance and/or circulating adiponectin levels.

## Methods

### Subjects

As previously reported, 63 patients (32 males and 31 females) diagnosed with nonalcoholic fatty liver disease were recruited from the outpatient clinic at the Wolfson Medical Center to participate in the study [7]. The study was a randomized, placebo controlled. The diagnosis of NAFLD was based on the results of abdominal ultrasonography and exclusion of viral, autoimmune or drug induced liver disease as well as alcohol intake of more that 20 g/day. Fatty liver disease was diagnosed on the basis on four sonographic criteria: (1) a diffuse hyperechoic echotexture (bright liver), (2) increased echotexture compared with the kidneys, (3) vascular blurring, and (4) deep attenuation [22].

Screening procedures included physical examination, complete blood chemistry, complete blood count, urinalysis and electrocardiography.

Patients with a history of unstable angina, myocardial infarction, cerebrovascular accident or major surgery within the 6 months preceding the study were excluded. Patients with unbalanced endocrine disease or any disease that might affect absorption of medications were excluded, as were patients with plasma creatinine >1.5 mg/dl, elevation of liver enzymes to more that twice the upper normal limit or electrolyte abnormalities (plasma potassium levels >5.5 mg/dl). Patients included in the study were stabilised on optimal medical treatment in the outpatient clinic for up to 3 months, and an effort was made not to change treatment during the study. Patients unbalanced during the 3 month run-in period were not included in the study. All concomitant medications were kept stable to prevent possible effects on the study variables. The patients were instructed to consult the study physician if any change in medical treatment was suggested by another physician. None of the study participants had previously received metformin therapy.

Patients were randomly assigned to one of two groups: Group 1 received oral daily metformin at a dose of 850–1700 mg/day; Group 2 received matching placebo capsules. Of the 63 patients recruited to the study, 52 completed the 4-month treatment period (27 from Group1 and 25 from Group 2). And 41 completed the 12-month treatment period (19 from Group 1 and 22 from Group 2). Metformin therapy was generally well tolerated; only one patient from metformin group withdrew because of gastrointestinal side effects during initial 4 month and 1 patient during additional 8 months. Additionally, 1 patient from metformin group did not return for follow-up because prolonged hospitalization for respiratory infection. In control group 2 patient discontinued follow-up because hospitalization due to urinary tract infection and elective hospitalization for cholecystectomy. The other participants who dropped out from the study were classed as ‘lost to follow-up’.

### Informed consent

The study was approved by the Institutional Review Board and the patients signed a full informed consent before participation. The study had been registered in ClinicalTrials.gov registry. The registration number: NCT01084486.

### Biochemical parameters

Blood sampling for full chemistry and metabolic parameters, including fasting glucose, fasting insulin, lipid profile, hs-CRP, liver function tests and plasma adiponectin was performed at baseline, after 4-month treatment period and at the end of the study. Glucose was measured using the Aeroset chemistry system (Abbott Diagnostics), high-density lipoprotein cholesterol (HDL), and triglycerides were assayed using an Aeroset automated analyzer (Abbott Diagnostics, Berkshire, UK), low-density lipoprotein cholesterol (LDL) was calculated using Friedewald’s formula and insulin was measured using an immunometric assay specific for human insulin (Invitron, Monmouth, UK). Adiponectin was determined by a commercial sandwitch enzyme immunoassay technique, R&D Systems, Minneapolis, USA (catalog number DRP300) with 2.8% intra-assay and 6.5% inter-assay variability.

Homeostasis model assessment-insulin resistance (HOMA-IR) was calculated by the following formula: fasting plasma insulin (mU/ml) x fasting plasma glucose (mg/dl)/405.

### Blood pressure and pulse wave velocity measurement

Blood pressure (BP) was measured using an automated digital oscillometric device (Omron model HEM 705-CP, Omron Corporation, Tokyo, Japan) and a mean of three readings was taken. The radial pressure waveform was recorded and subsequently transformed by using a validated generalized transfer function incorporated in the SphygmoCor (version 7.1, AtCor Medical, Sydney, Australia) to give an estimate of the corresponding central ascending aortic pulse wave. With the integral software, the central augmented pressure was calculated as the difference between the early and late systolic peaks of the estimated central pressure waveform. Central aortic augmentation index (AI) was calculated as the augmented pressure expressed as a percentage of the pulse pressure.

Arterial stiffness was determined at baseline visit at the end of 4-month and 12-month treatment period.

#### Statistical analysis

Analysis of data was carried out using SPSS 10.0 statistical analysis software (SPSS Inc., Chicago, IL, USA). For continuous variables, such as hemodynamic, biochemistry and arterial elasticity parameters, descriptive statistics were calculated and reported as mean ± standard deviation. Normalcy of distribution of continuous variables was assessed using the Kolmogorov-Smirnov test (cut off at *p* = 0.01). Categorical variables such as sex and co-morbidities were described using frequency distributions and are presented as frequency (%). Continuous variables were compared by treatment group using the *t*-test for independent samples. Additionally, univariate general linear modeling (GLM) was used to compare outcomes by treatment assignment controlling for baseline values of covariates. Within a given treatment group, the *t*-test for paired samples was used to compare before vs. post-treatment values of outcomes. Categorical variables were compared between groups using the chi-square test. All tests are two-sided and considered significant at *p* < 0.05.

## Results

The clinical characteristics of the study groups are presented in Table 1. As can be seen, there were no significant differences between the two groups in terms of age, sex, presence of cardiovascular risk factors, baseline blood pressure level, baseline blood pressure level, liver function and arterial stiffness. Concomitant medications were distributed similarly in both groups at the start and end of the study. The two variables, fasting glucose and serum adiponectin levels, differed significantly by groups at baseline. There were no notable differences in the participant characteristics at entry into the study between those who attended the final data-collection visit and the individuals who did not.

**Table 1:** Demographic and clinical characteristics of study patients

**Variables**	**Patients received metformin (n = 32)**	**Placebo group (n = 31)**	**p-value**
Male/Female	17/15	14/17	0.352
Age (y)	51.9 ± 10.9	55.2 ± 14.0	0.306
BMI (kg/m²)	32.6 ± 5.8	31.5 ± 5.6	0.410
Current smokers, n (%)	4 (12.5%)	2 (6.5%)	0.391
Hypertension, n (%)	15 (46.9%)	15 (48.4%)	0.599
Dyslipidemia, n (%)	21 (65.6%)	14 (45.2%)	0.079
DM/IFG, n (%)	6/14(18.8/43.8%)	2/13 (6.5/41.9%)	0.128
Concomitant medication:			
Antidiabetic treatment (%)	6 (18.8%)	2 (6.5%)	0.194
Statins (%)	18 (56.3%)	11 (35.5%)	0.079
ACEIs/ARBs (%)	9 (28.1%)	9 (29.0%)	0.609
Diuretics (%)	1 (3.1%)	6 (19.4%)	0.104
B-blockers (%)	9 (28.1%)	10 (32.3%)	0.784
CCB-blockers (%)	5 (15.6%)	8 (25.8%)	0.351
Aspirin (%)	13 (40.6%)	9 (29.0%)	0.292
Baseline systolic BP (mm/Hg)	138.8 ± 17.6	133.6 ± 18.1	0.258
Baseline diastolic BP (mm/Hg)	80.1 ± 9.8	74.6 ± 13.5	0.072
Baseline heart rate (bts/min)	68.6 ± 12.3	62.9 ± 8.4	0.034
Baseline fasting glucose (mg/dl)	131.8 ±51.3	98.3 ± 14.8	0.001
Baseline total cholesterol (mg/dl)	184.5 ± 43.3	191.1 ± 37.0	0.523
Baseline LDL Cholesterol (mg/dl)	110.4 ± 37.9	112.7 ± 34.2	0.811
Baseline HDL-cholesterol (mg/dl)	42.6 ± 13.0	48.2 ± 15.2	0.123
Baseline triglycerides (mg/dl)	185.5 ± 112.0	143.1 ± 63.2	0.070
Baseline hs-CRP (mg/dl)	0.9 ± 1.1	0.9 ± 1.4	0.969
Baseline AST (U/l)	28.9 ± 16.8	28.0 ± 8.2	0.796
Baseline ALT (U/l)	38.0 ± 29.9	32.6 ± 15.3	0.377
Baseline ALP (U/l)	67.7 ± 17.0	72.2 ± 23.5	0.386
Baseline creatinine (mg/dl)	0.9 ± 0.1	0.9 ± 0.2	0.723
Baseline urea (mg/dl)	32.0 ± 10.0	32.0 ± 9.0	1.000
Baseline adiponectine (ng/ml)	6130.5 ± 2872.6	9156.3 ± 6365.2	0.020
Baseline AI (%)	31.4 ± 11.1	30.0 ± 10.7	0.628

### Changes in hemodynamic, arterial stiffness and metabolic parameters in metformin treated patients

As can be seen in Table 2, both groups were similar at baseline in terms of hemodynamic and arterial stiffness parameters. Systolic (SBP) as well as diastolic blood pressure (DBP) were similar in both groups at baseline as well at the end of the study. AI decreased significantly during the treatment period: from 31.4+/−10.2 to 23.1+/−8.5% after 4 month (*p* < 0.0001) and 24.8 ± 7.9% after 12 month treatment period (*p* = 0.009).

**Table 2:** Changes in hemodynamic, arterial stiffness and metabolic parameters in metformin treated patients during 12 months follow up

**Variable**	**Metformin treated patients**				
	**Baseline**	**4 month**	**p-valueV1 vs V2**	**12 month**	**p-valueV1 vs V3**
Systolic BP (mm/Hg)	139.0 ± 17.9	137.2 ± 20.1	0.565	131.5 ± 14.0	0.060
Diastolic BP (mm/Hg)	80.6 ± 9.2	78.4 ± 9.0	0.403	80.9 ± 8.8	0.921
Aortic AP (mm/Hg)	15.6 ± 9.1	11.4 ± 7.6	0.001	12.8 ± 5.2	0.257
AI (%)	31.4 ± 10.2	23.1 ± 8.5	<0.0001	24.8 ± 7.9	0.009
Total Cholesterol (mg/dl)	179.3 ± 40.3	176.7 ± 32.2	0.722	178.2 ± 25	0.985
Triglycerides (mg/dl)	195.4 ± 119.1	157.9 ± 97.4	0.033	195.7 ± 149.7	0.574
HDL-cholesterol (mg/dl)	41.3 ± 12.2	45.7 ± 15.0	0.001	42.2 ± 11.6	0.768
LDL Cholesterol (mg/dl)	103.5 ± 37.3	103.7 ± 22.1	0.979	111.8 ± 23.8	0.286
ALP (U/l)	66.5 ± 17.8	61.1 ± 15.6	0.007	65.2 ± 19.7	0.452
ALT (U/l)	38.5 ± 31.7	29.3 ± 16.2	0.092	39.2 ± 21.8	0.838
AST (U/l)	29.0 ± 18.0	25.4 ± 9.7	0.300	30.6 ± 11.6	0.991
hs-CRP (mg/dl)	1.0 ± 1.1	0.5 ± 0.4	0.081	0.7 ± 0.9	0.448
Fasting glucose (mg/dl)	135.0 ± 52.8	115.8 ± 27.0	0.036	125.7 ± 54	0.185
Adiponectin (ng/ml)	5661.8 ± 2820	6166.6 ± 3265	0.171	6102.4 ± 2139	0.746
HOMA-IR	7.2 ± 6.3	7.3 ± 10.9	0.920	5.7 ± 6	0.042

As shown in Table 2, significant declines in fasting glucose, triglycerides and alkaline phosphatase (ALP) together with a significant increase in HDL cholesterol were observed during the initial 4 months in metformin treated patients. CRP and ALT decreased marginally during 4-month treatment period. However, after 4 months, there was no further improvement in fasting glucose and HDL cholesterol and there was gradual rise in ALP and ALT to the pretreatment levels. Insulin resistance assessed by HOMA-IR decreased significantly during 12 month treatment period. Serum adiponectin level tended to increase during treatment period with metformin; however, this increase did not reach statistical significance.

### Changes in hemodynamic, arterial stiffness and metabolic parameters in placebo group

As shown in Table 3, AI did not change significantly during the initial 4 month treatment period. Moreover, AI increased marginally after 12 months of the study (*p* = 0.089). No change was detected in either SBP or DBP during 12 month follow-up period. ALT, AST and serum adiponectin levels did not change in placebo group during the study.

**Table 3:** Changes in hemodynamic, arterial stiffness and metabolic parameters in control group during 12 months follow up

**Variable**	**Placebo group**				
	**Baseline**	**4 month**	**p-value V1 vs V2**	**12 month**	**p-value V1 vs V3**
Systolic BP (mm/Hg)	133.0 ± 19.5	139.8 ± 19.3	0.091	125.4 ± 25.5	0.477
Diastolic BP (mm/Hg)	73.3 ± 14.1	75.0 ± 9.4	0.589	73.5 ± 11.9	0.842
Aortic AP (mm/Hg)	15.6 ± 9.3	20.2 ± 18.6	0.271	17.2 ± 12	0.587
AI (%)	29.9 ± 11.0	29.0 ± 11.5	0.625	34.6 ± 12.1	0.089
Total Cholesterol (mg/dl)	190.0 ± 38.5	186.2 ± 32.6	0.616	175.8 ± 30.6	0.024
Triglycerides (mg/dl)	140.8 ± 58.0	135.0 ± 57.6	0.588	146.2 ± 70.5	0.447
HDL-cholesterol (mg/dl)	47.8 ± 14.3	49.6 ± 15.3	0.329	49 ± 15.9	0.278
LDL Cholesterol (mg/dl)	110.8 ± 35.2	109.0 ± 25.7	0.786	103.3 ± 27.2	0.521
ALP (U/l)	74.3 ± 24.6	67.4 ± 19.6	0.065	71.5 ± 22.9	0.659
ALT (U/l)	34.9 ± 15.8	29.7 ± 16.3	0.120	32.1 ± 20.6	0.315
AST (U/l)	29.4 ± 8.4	27.4 ± 8.3	0.246	29.3 ± 12.9	0.957
hs-CRP (mg/dl)	1.0 ± 1.5	0.5 ± 0.5	0.139	0.5 ± 0.7	0.156
Fasting glucose (mg/dl)	98.1 ± 15.9	103.9 ± 18.5	0.015	98 ± 14.2	0.651
Adiponectin (ng/ml)	10251.8 ± 6610	10020.2 ± 5648	0.699	10255.8 ± 5704.4	0.529
HOMA-IR	5.4 ± 4.9	4.9 ± 7.5	0.682	4.97 ± 8	0.721

### Between group comparisons

**Figure 1:**
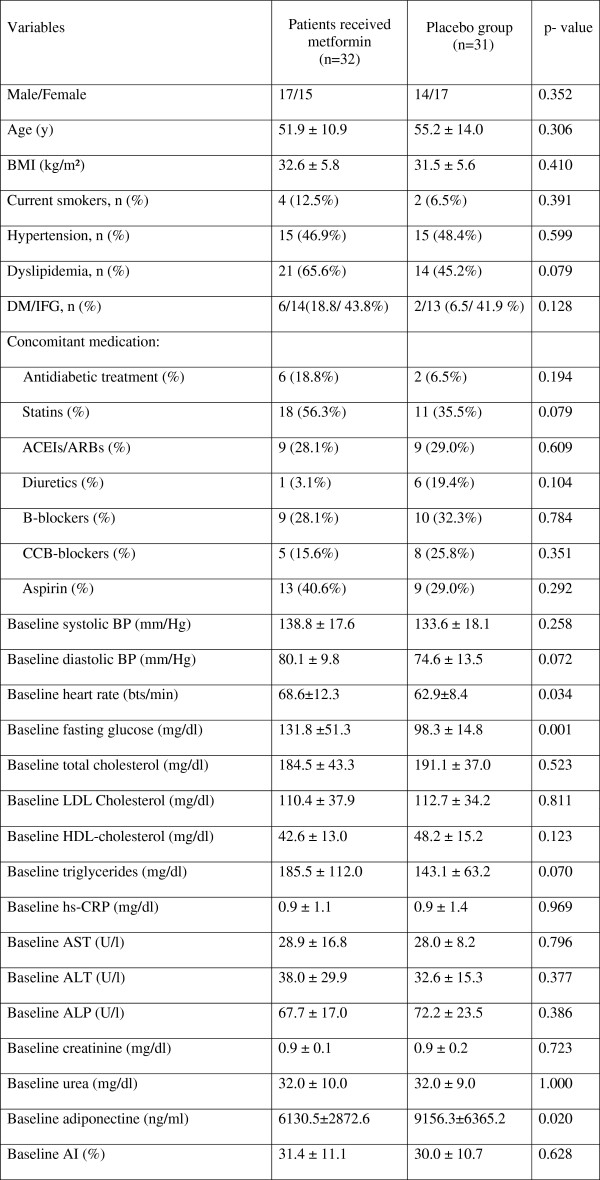
**Augmentation Index by groups during 12-month follow-up.** * p-value < 0.05, ** p-value < 0.01, *** p-value < 0.001. Values are means ± SD.

As can be seen in Figure 1, AI did not differ significantly between the groups at baseline. However, AI was significantly lower in patients treated with metformin than in the placebo group after initial 4 months (*p* = 0.038) as well as at the end of the study (*p* = 0.021). During one year follow up AI decreased by 7.47% in the active treatment group and increases by 4.45% in the control group (*p* = 0.004). Because fasting glucose and serum adiponectin levels differed significantly by groups at baseline, univariate GLM analysis was carried out to control for these findings. Baseline mean arterial pressure (MAP), as representative of hemodynamic values was also included as a covariate in this model. Significant by-group differences in Δ AI (change from baseline AI) persisted even after adjustment for baseline values of MAP, fasting glucose and serum adiponectin levels (*p* = 0.004).

### General linear model

In this repeated measures model, the pattern of change in fasting glucose and adiponectin over time was significantly different between metformin treated patients and controls (*p* = 0.004 and *p* = 0.001, respectively). The pattern of change in HOMA-IR over time did not differ by treatment group.

To determine whether the change in arterial stiffness result from improved glycemic control per se in active treatment group or from other factors such as exposure to metformin, change in insulin resistance or adiponectin levels, general linear model of change from baseline AI (Δ AI) was carried out. In this model change from baseline glucose, adiponectin levels, HOMA-IR as well as age, sex, baseline mean arterial pressure, baseline AI were included. Additionally, metformin exposure was included as a fixed factor. The model was significant (*p* = 0.006) and explained 47.5% of the variability in this outcome. Significantly associated with ΔAI were baseline AI (*p* = 0.001), exposure to metformin (*p* = 0.037) and change from baseline adiponectin levels (*p* = 0.032). Change from baseline glucose was not associated with the outcome (*p* = 0.709) (Table 4).

**Table 4:** General liner model of AI

**Source**	**Type III Sum of Squares**	**Mean Square**	**F**	**Sig.**
Corrected Model	1953.803(a)	195.380	3.899	0.004
Age	151.237	151.237	3.018	0.096
Sex	51.227	51.227	1.022	0.323
Δ Fast. Glucose	7.181	7.181	0.143	0.709
ΔHBA1C	7.083	7.083	0.141	0.711
Δ HOMA-IR	49.744	49.744	0.993	0.330
Δ Adiponectin	263.214	263.214	5.253	0.032
Δ MAP	10.152	10.152	0.203	0.657
Group	247.677	247.677	4.943	0.037

## Discussion

The present randomized, placebo controlled study demonstrates that one year metformin treatment was associated with a significant improvement in arterial stiffness in patients with nonalcoholic fatty liver disease. This beneficial vascular effect was associated with exposure to metformin per se as well as changes in circulating adiponectin levels.

Previous clinical and experimental data show the beneficial vascular effect of metformin. Metformin treatment increases insulin action in peripheral tissue, resulting improvement in endothelium-dependent vasodilatation [23], decrease in the local activity of growth factors in vascular tissue, which in turn decrease a development of vascular smooth muscle cell hypertrophy [9,11,24]. It has been shown that metformin has beneficial effect on nitroxidation, endothelial function and IMT in patients with metabolic syndrome [25] and significantly improves arterial stiffness and endothelial function in young women with polycystic ovary syndrome [26]. In addition, previously published data indicate that metformin treatment decreases matrix metalloproteinase (MMP)-9 in drug naive diabetic patients [27] and improves endothelial function in patient with type 2 diabetes as well as first-degree relatives of type 2 diabetic patients [9,28]. We have previously reported that 4 months metformin treatment was associated with a significant improvement in arterial stiffness in patients with nonalcoholic fatty liver disease [7]. This beneficial vascular effect was accompanied by an improvement in glucose and lipid metabolism as well as liver enzymes such as ALP and ALT. Although the anti-atherogenic effect of short term metformin has been assessed previously, data regarding the long-term impact of metformin on arterial stiffness are limited. In the present study, investigating vascular effect of metformin treatment during one year, we observed impressive improvement in arterial stiffness in patient with NAFLD treated with metformin. Moreover, marginal increase in AI has been observed in the placebo group during 12 month follow up. These vascular changes can be conceptualized as the “natural” development expected in untreated individuals with NAFLD. This means that we would have expected an increase in arterial stiffness over time in subjects with hepatic steatosis, but treatment by a potent insulin sensitizer not only prevented the “natural” increase, it reversed the anticipated process and produced an improvement in arterial stiffness during a relatively short (4 months) as well as long-term (one year) period of time.

Another important observation in our study is the long term effect of metformin treatment on liver function in patients with NAFLD, since data regarding the impact of metformin on hepatic steatosis are limited and controversial. Metformin have been shown to activate AMP-activated protein kinase (AMPK) in hepatocytes as well as phosphorylation of acetyl-CoA carboxylase, resulting in decreasing hepatic lipogenesis [23]. It has been demonstrated that the liver protective mechanisms of metformin in non-alcoholic fatty liver disease may be contributed to down-regulation of secretory phospholipase A2 mRNA expression, decrease in serum secretory phospholipase A2, lysophosphatidylcholine and lower inflammatory response in rat model [29] Although animal data in experimental models have demonstrated that this treatment is able to retard the progression of hepatosteatosis, results from human studies have been varied. Studies in pediatric subjects with NAFLD show that metformin wasn't superior to placebo in attaining the primary outcome of sustained reduction in ALT level [30]. On the other hand, previously published data from the Diabetes Prevention Program, have demonstrated that serum ALT activity as a marker for NAFLD was consistently lower in those treated with metformin compared with placebo during 3 year follow-up. However, this effect of metformin therapy on ALT was mediated by weight loss [31]. Additionally, the effect of metformin was tested in subjects with NASH consuming metformin or lipid and calorie-restricted diet for 6 months. Liver enzyme activities as well as insulin sensitivity improved in the metformin-treated patients, while no significant differences in necroinflammatory activity and fibrosis were found between the groups [32]. In the present study a significant decrease in alkaline phosphatase and marginal decrease in ALT were observed during the initial 4 months in metformin treated patients. However, after 12 months of treatment, a gradual rise in ALP and ALT to the pretreatment levels was observed.

The present study demonstrated that beneficial effect of long-term metformin treatment on arterial stiffness was associated with change in circulating adiponectin, a collagen-like protein specifically expressed in human adipose cells which plays an important role in insulin sensitivity, inflammation and atherogenesis [33,34]. Recently adiponectin has been considered as a possible link between liver dysfunction and atherosclerotic vascular disease in patients with NAFLD [35]. In the present study, we did not observe significant changes in circulating adiponectin levels, however serum adiponectin level tended to increase in metformin treated patients during the study.

Moreover, the pattern of change in adiponectin over time was significantly different between metformin treated patients and controls. Additionally, in general linear model change from baseline adiponectin was significantly associated with ΔAI.

The findings of the present study suggest that circulating adiponectin level affects arterial stiffness more strongly than insulin resistance per se. Nevertheless, further studies are needed to clarify the clinical effects of the circulating adiponectin on arterial stiffness.

Our study has several limitations. Our study has focused on patients with nonalcoholic fatty liver disease, therefore, the application of our findings to other patient populations remains uncertain. Since the present study includes a small number of participants and a relatively large number of dropouts, additional studies are required to establish the beneficial vascular effect of metformin as well as its clinical impact on cardiovascular outcomes in subjects with nonalcoholic fatty liver disease. Moreover, because serum levels of total adiponectin were assayed in the present study, the impact of different multimers of adiponectin (low, middle and high molecular weight form) on vascular pathophysiology remains unclear.

## Conclusions

We have demonstrated that one year metformin treatment improves augmentation index in patients with nonalcoholic fatty liver disease. These beneficial vascular effects was associated with exposure to metformin per se as well as change in circulating adiponectin levels, suggesting that metformin may mediate its vascular effects via glicemic control-independent mechanisms.

## Competing interests

The corresponding author and all of the authors have no conflicts of interest or financial or other contractual agreements that might cause conflicts of interest.

## Authors’ contributions

MS and DG contributed to the study conception and design. EO was responsible for the data acquisition. MB performed the analysis and interpretation of data. ZM carried out the immunoassays. MS was responsible for review the existing literature and for writing the first draft of the paper. All authors performed a critical revision of the manuscript for important intellectual content. All authors read and approved the final manuscript.

## Funding

This research did not receive any specific grant from any funding agency in the public, commercial or not-for-profit sector.
